# Visual Trails: Do the Doors of Perception Open Periodically?

**DOI:** 10.1371/journal.pbio.1001056

**Published:** 2011-05-10

**Authors:** Julien Dubois, Rufin VanRullen

**Affiliations:** 1 Université de Toulouse, Centre de Recherche Cerveau et Cognition, Université Paul Sabatier, Toulouse, France; 2 Centre National de la Recherche Scientifique, UMR5549, Faculté de Médecine de Purpan, Toulouse, France

“Visual trailing” is a transient but dramatic disturbance of visual motion perception of unknown origin: the subject perceives a series of discrete stationary images trailing in the wake of otherwise normally moving objects. Although this phenomenon is most frequently encountered after ingestion of prescription and/or illicit drugs (most commonly with lysergic acid diethylamid, or LSD), it has also occasionally been reported following brain damage or neurological disorders. A quantitative account of visual trails is lacking; we argue that careful experimental investigation could potentially reveal how our brains update conscious visual perception in time.

## What Do Visual Trails Look Like?

**Figure 1:**
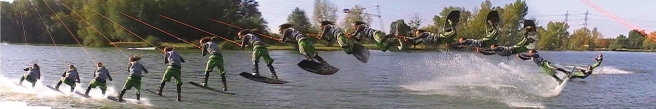
Multiple-exposure stroboscopic photograph. This illustrates (inasmuch as possible with a static image) the perception experienced during trailing.

Ask any LSD user: they know the drug is taking effect when the “good trails” kick in. Trailing is a visual perceptual effect commonly experienced during LSD consumption and as a long-lasting side effect of the drug (hallucinogen persisting perception disorder) [1]–[4]. LSD users perceive a series of discrete positive afterimages in the wake of moving objects, a percept that has been likened to a multiple-exposure stroboscopic photograph, somewhat akin to Etienne-Jules Marey's chronophotographs [5] from 1880, or to more recent digital art produced in a few clicks (Figure 1).

Trailing is a visual disturbance that has been observed under various conditions. While some authors use the terminology of LSD users [1]–[3],[6],[7], others describe the phenomenon in more clinically suited terms: examples are “akinetopsia” [8],[9], “polyopia”[9], “palinopsia” [4],[10]–[13], or “visual perseveration” [14]. In Table 1, we clarify the meaning of each of these terms (according to [5],[8],[15]–[21]). However, none of these terms precisely captures the specific elements of visual trails: they occur only for moving objects (unlike polyopia); moving objects are still perceived as moving (unlike akinetopsia); duplicate images are perceived only in the presence of real objects (unlike palinopsia); and, most importantly, visual trails are not mere streaks ([22]), as would appear on a long-exposure photograph of moving objects (visual perseveration). Rather, trailing consists of a discrete series of snapshots of the moving object along its past trajectory, as if successive frames of a movie had been superimposed. These phenomenological features of trailing are clearly portrayed in the case reports that we collected (Box 1). The discreteness of the trailing percept, in particular, is one of its most characteristic, and most mysterious, aspects.

**Table 1:** Glossary.

TRAILING	An object in motion is followed by a discrete series of stationary images that slowly fade out along its trajectory	
	Our Definition	How It Differs from Trailing
AKINETOPSIA	An object in motion is not perceived as moving continuously; rather, it is experienced as a series of stills	The perception of motion is abolished and a single frame can be seen at any one time
POLYOPIA	Multiple images of an object (from two to 100 in extreme cases) are perceived simultaneously	Polyopia is not specific to objects in motion
VISUAL PERSEVERATION	A positive afterimage persists after removal of an object	Visual perseveration for a moving object would leave a blur in its wake, rather than discrete images
PALINOPSIA	Similar to visual perseveration; some authors describe a lag between the removal of the object and the onset of the afterimage	Same as visual perseveration, potentially with a gap between the moving object and the perseverant tail

**Box 1. Selected Case Reports**

**Lysergic acid diethylamide (LSD)**

“[When asked] if they ever ‘saw any trails’, [the subject] would wave a hand across the visual field and say ‘Oh, you mean these?’ The subject could then describe a trail of images of the moving hand, much like the frames of a piece of motion picture film frozen in space long enough for the subject to see them” [3].

**Nefazodone**

“A 47-year-old man reported seeing streams of multiple, frozen images trailing in the wake of moving objects. As soon as motion ceased, the images collapsed into each other. He compared his vision to a scene lit by a flashing strobe, except that stationary elements were perceived normally. In fact, if nothing was in motion and he held perfectly still, his vision was entirely normal. The moment anything moved, however, it left a stream of static copies in its path. For example, while out for an evening stroll, he saw a pack of identical dogs lined up behind his West Highland terrier. Driving was impossible because he was confused by multiple snapshots of cars, streets, and signs. Moving lights were followed by a long comet tail” [8].

**Trazodone**

“He began to have numerous morning episodes of ‘strobe images’. They were most apt to occur in dim illumination. He described looking at the door of his bedroom and seeing multiple images of the door ‘march’ across his field of vision when he shifted his gaze to the bureau on the other side of the room. The false images were intense, and he had many such episodes every morning, each lasting 15 minutes or less” [10].

**Mirtazapine**

“As she watched her husband walk past her, she saw multiple afterimages of him as if he were leaving a visual trail. These afterimages were less color intensive than the normal visual image, slightly blurred, and faded away after 30 seconds to 1 minute. The phenomenon repeated itself with most moving objects and was generally more pronounced with objects in Ms. A's lateral visual fields” [29].

**Topiramate**

“Ms. A reported seeing ‘picture in picture’ images, like she was in a ‘discotheque’, or in a place with stroboscopic lights. Those persistent ‘frozen pictures’ faded away after a few seconds. The phenomenon repeated itself with most moving objects” [11].

## Who Experiences Visual Trails?

While LSD induces trailing [1]–[3],[23], consumption of other hallucinogens like psilocin and mescaline has not been linked with such visual disturbances in the clinical literature (to our knowledge). Either their interaction with the receptors that mediate trailing is not as potent, or LSD acts at sites that other hallucinogens do not bind with (for a review of the pharmacology of hallucinogens, see [24] and for LSD more specifically, see [25],[26]). Reports pertaining to the past use of illicit drugs are, however, poorly controlled (a good example is a report of illegal marijuana leading to trailing by Levi and Miller [27], in which they explicitly acknowledge that their cases may be confounded by the presence of impurities in illegal marijuana, as well as by past use of LSD).

A better understanding of the pharmacology of trailing may arise from the report of similar visual disturbances following the use of prescription drugs. The chemistry and dosage of prescription drugs is well controlled, which is not the case for illicit drugs. Nefazodone, for instance, is a recent antidepressant drug with multiple reports of episodes of visual trails as a side effect [6]–[8],[28]. A closely related antidepressant drug, trazodone, can induce similar side effects [10]; so can mirtazapine [29], and, possibly, risperidone (the study is inconclusive due to the concurrent administration of trazodone [30]). These four antidepressant drugs all antagonize 5-HT2a and 5-HT2c receptors, and it has been proposed that this is at the origin of the trailing phenomenon [28]. However, the first recognized inducer of visual trails, LSD, has the opposite effect (agonist activity) at both these receptors [24]–[26]. Common to these antidepressant drugs and LSD is an increase in extracellular serotonin levels (indeed, all these drugs have been linked to serotonin syndrome [31], a form of poisoning due to excess serotoninergic activity that causes a range of symptoms, including hallucinations, elevated body temperature, and fast heart beat). Although the evidence is suggestive of a link between serotonin and visual trailing, the widespread action of serotonin throughout the brain prevents us from speculating further for the time being.

Visual trails have also been experienced with other drugs with very different pharmacology. The antiepileptic drug topiramate, which, among other actions, blocks voltage-dependent sodium channels and increases GABA activity at some GABA-A receptor subtypes, has been involved with visual trails [11],[12]. Increased inhibition, mediated by higher levels of serotonin or GABA potentiation, stands out as a likely common action of this drug and the ones described previously. Topiramate also inhibits cytochrome isoform CYP2D6 [32], which has been associated with serotonin syndrome [31].

Besides pharmacologically induced trailing, some clinical reports from neuropsychological populations deserve to be mentioned. A crisp account of migraine patients experiencing trailing is given by Sacks [5], where he estimates the frequency of snapshots at “six to twelve frames per second” (see also [33]). We found one other report of visual trails (combined with akinetopsia and palinopsia) in migraineurs [34] (see also medical doctor Klaus Podoll's Web site [35]). On the whole, trailing is a very rare disturbance for migraine sufferers, the migraine being a very heterogeneous condition that affects roughly 20% of the population at one time or another; the possibility exists that the disturbance occurs only for a very specific and rare form of migraine, or is a side effect of drugs that patients take to relieve their symptoms [12]. As a side note of interest, controlled studies have found migraine patients to have impairments for global motion perception [36] (see also [37]).

A recent case study [9] described visual trails associated with another neurological condition: the posterior cortical atrophy variant of Alzheimer's disease [38]. The symptoms are referred to as akinetopsia/polyopia by the authors, but their description corresponds to a direction-specific version of trailing (visual trails are perceived when objects move from right to left, but not when they move from left to right). Direction-specificity restricts the mechanistic models that can be put forward to explain visual trails; however, the etiology is very different from previously discussed cases, and in the absence of other reports of directional trailing, it is premature to draw conclusions from this report.

Finally, a very rare disturbance of motion perception, for which Zeki coined the word akinetopsia [15], can follow bilateral lesions in the occipito-temporo-parietal cortices [39]. Patient L. M. permanently lost motion perception (except for slowly moving objects [40]). Instead of seeing a moving object, she saw the object in a series of stills, as in a movie run too slowly. This is a rather extreme case that differs qualitatively from trailing (see Table 1), but it may ultimately rely on similar mechanisms.

## What May Cause the Perception of Visual Trails?

Visual trails, because of their discrete and repetitive nature, may represent the perceptual manifestation of an underlying periodic process. This periodicity could arise outside of the neural system (e.g., eye movements, motor tremor), or may be the result of faulty motion computation mechanisms (e.g., motion smear suppression), or, finally, may point to a more general, quasi-periodic sampling process that affects, among other things, the motion perception system. These three (non-exhaustive and non-exclusive) possibilities are developed below.

### Do Visual Trails Arise from Abnormal Eye Movements?

Trailing may not originate in the neural pathways at all. For example, if visual trails are only perceived when subjects track a moving object with their eyes, the discrete afterimages may be caused by a defective, jerky smooth-pursuit mechanism. Saccadic suppression would operate while the eyes execute repeated corrective saccades to keep track of the moving object, shutting on and off the visual input intermittently. Note that people having experienced trailing often recount that trails follow just one or a few moving objects at a time, rather than affecting the entire visual scene, as would be the case after eye movements (or uncontrolled tremors). This makes such a motor explanation unlikely; nonetheless, a well-controlled eye tracking experiment on individuals experiencing visual trails would be essential to evaluate this hypothesis.

### Do Visual Trails Reflect a Failure of Specific Motion Perception Mechanisms?

The periodicity may arise as a disturbance of certain motion processes that are normally continuous. For example, motion streak suppression [41] is an inhibitory mechanism allowing the brain to regulate the smear that a moving object leaves in its wake, owing to visible persistence (an image normally takes about 100 ms to fade from perception, long enough to blur the trajectory of a moving object as in a long-exposure photograph). Under the effects of LSD or related drugs, the streak suppression process might fall out of its normal operating range, resulting in stronger than normal local inhibition followed by excitatory rebound. This hypothesis would predict that trailing is accompanied by abnormal oscillatory activity confined within the motion perception system, a prediction that could be verified in human or non-human primates with appropriate recordings.

### Could Visual Trails Be the Manifestation of Periodicities Inherent to all Perceptual Processes?

Trailing may reflect a more widespread rhythmic activity affecting (possibly among other modules) the motion perception system. Two alternatives must be distinguished here, which could be teased apart by comparing oscillatory brain activity in the normal and drug-altered states. Either this rhythmic activity is directly produced by the drug (or at least, increased beyond perceptual threshold)—a rise of inhibition could generate prominent oscillations, turning the normally continuous processing of visual information into a series of discrete snapshots—or this periodicity is always present in the normal brain, but inaccessible to conscious perception. In the latter case, the drug may increase visible persistence, or disrupt motion streak suppression, two processes that would normally serve to hide the discrete visual trails.

## Related Phenomena Pointing to Periodicities in Visual Perception

A persistent thread in the perceptual literature is that temporal binning is, indeed, common to all of perception (reviewed by [42]). One of the most striking phenomenological manifestations of the discrete nature of perception is the so-called “continuous wagon wheel illusion”: in plain sunlight, a continuously rotating, spoked wheel can be perceived to rotate in a direction opposite to its true motion. While movie watchers are accustomed to this percept (which follows from an undersampling of the continuous motion of the wheel by the discrete frame rate of the camera and the movie projection system), perceiving reversals in conditions of continuous illumination is more challenging. Quasi-periodic sampling or binning processes within the visual system have been proposed as an explanation; this interpretation has been a subject of controversy, and many experiments have been conducted to test it. In light of these recent experiments, VanRullen and colleagues hypothesized that attentional processes may function in a quasi-periodic manner [43],[44]: when attention is deployed, it samples information at discrete moments in time. The rate of sampling may be dependent on the task at hand [43],[45],[46]. One component of this theory purports that the attentive motion system takes discrete samples of the object in motion to compute its trajectory. Past snapshots are usually concealed from conscious perception—but some substances may interfere with their proper suppression, giving rise to the trailing phenomenon.

## Towards a Solution: Measuring Visual Trails

How many “ghost images” [23] trail in the wake of the moving objects? How far apart are the “discrete and discontinuous images” [2]? How long do they persist for? These questions have, so far, not been answered. In fact, visual trails have been considered an annoyance at best, and quantifying them has not been the main concern in the various clinical settings in which they were observed. Careful quantitative investigation will be needed to shed light on the mechanisms that cause visual trails to appear as a discrete series of snapshots of the moving object.

As a preliminary step towards quantitative answers, we conducted an online survey in which we asked self-declared past LSD users to decide which of ten short movies with simulated visual trails best matched their recollected experience. We varied the interval between simultaneously perceived snapshots from 25 ms to 250 ms, keeping the number of concurrently visible repetitions to four. For over more than 210 participants, the responses were not randomly distributed across the ten choices, but followed a highly consistent pattern (chi-squared test, *p*<0.0008) with a preference for faster snapshot periodicities; participants selected a time interval between images of 67 ms on average, corresponding to an underlying periodicity in the 15–20 Hz (beta) range. Although this is the first study that tries to quantitatively assess the frame rate of LSD-induced visual trails, it suffers from numerous pitfalls. First, we have little control over the individuals who take the survey, as they do so anonymously from their home computer. However, this protocol follows a new trend in psychological research of using the Web for large-scale studies (see the http://www.testmybrain.org/ Web site recently created by Harvard scientists). Second, the responses rely on the memory of the percept, which may be faulty. Last, the chemical composition and dose taken by each individual is not controlled, which is problematic considering that more than 200 types of LSD tablets have been encountered since 1969 and more than 350 blotter paper designs have been in circulation since 1975 [47] (also, some blotters sold as LSD are in fact mimics). In fact, some of the most experienced users indicated that trailing is dose dependent. For all these reasons, one must be cautious in interpreting these initial results. The movie used for the survey as well as a results summary can be found online at http://www.cerco.ups-tlse.fr/~rufin/lsdsurvey.

Collecting further quantitative data with individuals who experience visual trailing will be necessary to tease apart the alternative accounts—possibly in combination with computational modeling. Solving the mystery of the origins of the trailing effect might reveal something deep about the mechanisms underlying perception, challenging the way we think we perceive the world.

## Abbreviations

LSD: lysergic acid diethylamid
